# Research during Pediatric Residency Training: A Nationwide Study in Japan

**DOI:** 10.31662/jmaj.2018-0007

**Published:** 2019-02-01

**Authors:** Akira Ishiguro, Osamu Nomura, Nobuaki Michihata, Tohru Kobayashi, Rintaro Mori, Katsumi Nishiya, Kazunari Kaneko, Japan Pediatric Society Steering Committee of Board Examination

**Affiliations:** 1 Center for Postgraduate Education and Training, National Center for Child Health and Development, Tokyo, Japan; 2 Department of Management and Strategy, Clinical Research Center, National Center for Child Health and Development, Tokyo, Japan; 3 Department of Health Policy, Research Institute, National Center for Child Health and Development, Tokyo, Japan; 4 Center for Medical Education, Kansai Medical University, Osaka, Japan; 5 Department of Pediatrics, Kansai Medical University, Osaka, Japan

**Keywords:** Research, pediatric training, residency program, education, board examination

## Abstract

**Introduction:**

Training in scholarship is an essential component of postgraduate education. Previous studies worldwide on the research activities of pediatric residents were questionnaires targeting program directors or surveys conducted in a limited number of institutions; however, no nationwide studies have been conducted. The objective of this study was to describe the research activities of pediatric residents.

**Methods:**

We conducted a nationwide cross-sectional study during 2015 and 2016 in Japan. Study data were collected from each resident’s logbook submitted to the board examination office and compared by the type of institution, namely, university, children’s, or community hospital.

**Results:**

Of 1,718 eligible participants, 1,500 participated in this study. Overall, 499 (33.3%) residents trained at national/public university hospitals, 371 (24.7%) at private university hospitals, 140 (9.3%) at children’s hospitals, and 490 (32.7%) at community hospitals. Although 1,361 (90.7%) residents gave at least one presentation at an academic conference during their residency, only 235 (15.7%) residents published one or more articles in a peer-reviewed academic medical journal. The proportion of residents who gave at least one presentation (*p*=0.03) and published at least one study (*p*<0.01) differed significantly among the types of institutions. Residents at community hospitals gave fewer presentations at conferences (odds ratio [OR] 0.56; 95% confidence interval [95% CI] 0.36–0.87) and published fewer peer-reviewed articles (OR 0.53; 95% CI 0.37–0.76) than residents at national/public university hospitals.

**Conclusions:**

This is apparently the first nationwide study demonstrating that the research activities of pediatric residents consisted mostly of presentations at academic conferences, but also that most residents had not published their research. There was a marked variation in residents’ academic activities by institution type.

## Introduction

Scholarship is an important activity in the career of any health professional. Scholarly activity has been recognized as an essential component of postgraduate medical education ^(1), (2)^. The Accreditation Council for Graduate Medical Education (ACGME) states that “residents should participate in scholarly activity.” Residency programs accredited by the ACGME must provide curricula designed to advance residents’ skills in research and opportunities for residents to participate in scholarly activities ^(3), (4)^. The Royal College of Physicians and Surgeons of Canada has also proposed the CanMEDs framework including seven competencies, one of which, named “scholar,” was defined as “physicians demonstrat(ing) a lifelong commitment to excellence in practice through continuous learning and by teaching others, evaluating evidence, and contributing to scholarship” ^(5)^.

In medicine, scholarship is concerned with applying the empirical findings available in previously published studies to a given local or particular context ^(6)^. The Japan Pediatric Society (JPS) adopted “scholar” as one of the five competencies needed to be a qualified pediatrician. Despite the awareness of the faculty and researchers on the importance of research activity in postgraduate education ^(7), (8)^, pediatric residents’ research activities have not been fully investigated anywhere. Previous research was limited to questionnaires targeting program directors and cross-sectional multicenter surveys ^(9), (10), (11), (12)^. Thus, a detailed, nationwide study investigating research activities during a pediatric residency is yet to be conducted. The objective of this study was to describe the current state of scholarly activities of residents in Japanese pediatric programs nationwide.

## Materials and Methods

### Setting: postgraduate pediatric education in Japan

The JPS started a board certification system ten years ago, and as a result, pediatric residency programs across Japan have recently begun to show an improvement in quality. A decade ago, not all pediatric trainees obtained board certification due to insufficient incentives. However, the system is now recognized nationally, and almost all pediatric residents take the examination for certification by the JPS. Moreover, experienced pediatricians who had not taken the board examination before have also come to recognize the value of JPS certification and have applied to take the test. As a result, candidates with a relatively wide range of experience now take the examination, including non-resident pediatricians who have already practiced for many years.

### Definitions of research activity, presentations, and publications

In this report, we defined “research activity” as research presentations at academic conferences or meetings and publications in academic journals. Research included case reports, experimental studies, and review analyses. Academic conferences/meetings referred to those held by academic societies; thus, conferences within residents’ institutions, such as case conferences, grand-rounds, and morbidity and mortality conferences, were not included. Academic journals included both peer-reviewed and non-peer-reviewed journals; however, peer-reviewed journals were mainly analyzed. For international journals, only PubMed-indexed journals were enrolled. Journals for laypersons were excluded.

### Design and participants

The JPS reformed the board examination system in 2017 and included the experience of writing a research paper (e.g., a case report, an original article, or a review article) in the eligibility criteria for the examination. As such, we conducted a nationwide cross-sectional study in 2015 and 2016 in Japan to ascertain the research activities of pediatric residents prior to the reforms, that is, when no research activities were required regardless of the program type (i.e., academic or not). All pediatric residency trainees in Japan who took the pediatric board examinations during those two years participated in this study. Pediatricians who declined participation in this study were excluded. We further excluded pediatricians with ten or more postgraduate years since these physicians had, in many instances, practically achieved the level of attending physician and were unsuitable candidates for our investigation of scholarly activities among pediatric residents.

### Data collection

Study data were collected from each resident’s logbook submitted to the office of the JPS and the board examination results. Information including the name of the institution, record of research presentations (time of the presentations and type of event), and record of research papers (number and type of papers) was extracted by trained analysts.

### Analysis

We employed descriptive statistics to characterize participants by duration of training, type and location of their institution, experience in research (presentations at conferences and/or publications), and the number of publications and presentations given at conferences. The frequencies and percentiles were calculated for the variables. Institutions were divided into university hospitals, children’s hospitals, and community hospitals. We defined a children’s hospital as a hospital belonging to the Japanese Association of Children’s Hospitals and Related Institutions (JACHRI), excluding university hospitals. The definition of community hospital was a non-university hospital that was not a member of the JACHRI. University hospitals were private hospitals and national or public hospitals were operated by the national or local government, respectively. Results were expressed as the mean (± standard deviation [SD]), unless otherwise specified. Categorical variables were compared by the chi-squared test, and the characteristics of the institutions were compared by one-way analysis of variance (ANOVA). Bonferroni’s multiple comparison was applied if the results of ANOVA were statistically significant. Multivariate logistic regression analysis was conducted to identify factors associated with the presentation and publication experience of residents using the duration of training and type and location of the institutions. Data were analyzed using STATA 15 (STATA Corporation, College Station, TX, USA).

### Ethical aspects

This study was approved by the Ethics Committees of both the National Center for Child Health and Development in December 2014 (No. 74) and the JPS in March 2015.

## Results

**Figure 1.**
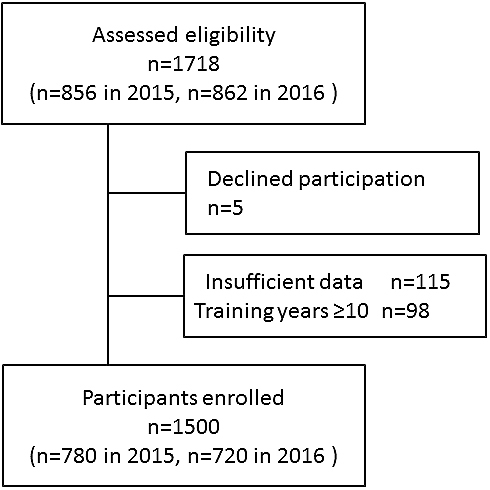
Flow chart of this study.

Of 1,718 eligible participants, 218 were excluded and 1,500 residents were included (Figure 1). The mean duration of their residency training was 3.9 (±1.4) years. Of all the participants, 499 (33.3%) trained at a national/public university hospital, 371 (24.7%) at a private university hospital, 140 (9.3%) at a children’s hospital, and 490 (32.7%) at a community hospital. The geographical distribution of the participants was as follows: 371 (24.7%) were located in Tokyo, 224 (14.9%) in Kanto excluding Tokyo, 271 (18.3%) in Kinki, 215 (14.3%) in Chubu, 186 (12.4%) in Kyushu/Okinawa, 79 (5.3%) in Tohoku, 65 (4.3%) in Chugoku, 48 (3.2%) in Hokkaido, and 38 (2.5%) in Shikoku.

Almost all residents (1,361, 90.7%) gave at least one presentation at an academic conference during their residency (Table 1). On average, they gave 4.4 (±3.1) presentations during their residency. Regarding publications, only 327 (21.8%) residents published one or more articles in an academic medical journal. They commonly submitted to a peer-reviewed journal (235, 15.7%), and 142 (9.5%) residents published articles in a non-peer-reviewed journal. Among peer-reviewed journals, they submitted to a Japanese domestic journal (179, 11.9%), and only 73 (4.9%) residents submitted to an international journal. While many of the residents had written a case report (177, 11.8%), 72 (4.8%) had written an original article. On average, they published 0.3 (±1.5) articles during their residency.

**Table 1.** Research Experience.

	Number of experiences (%)	Number of presentations or publications (mean ± SD)
Presentations at academic conferences		
Presentations at any academic conference	1361 (90.7)	4.4 ± 3.1
Presentations per year at any academic conference	NA	1.2 ± 0.9
Publications in academic journals		
Publications in any academic journal	327 (21.8)	0.3 ± 1.5
Publications in peer–reviewed journals	235 (15.7)	0.2 ± 0.6
Peer–reviewed Japanese domestic journals	179 (11.9)	0.1 ± 0.5
Peer–reviewed international journals	73 (4.9)	0.1 ± 0.3
Peer–reviewed case reports	177 (11.8)	0.1 ± 0.5
Peer–reviewed original articles	72 (4.8)	0.1 ± 0.3
Publications in non–peer–reviewed journals	142 (9.5)	0.1 ± 1.1

NA, not available.

The proportion of residents who gave at least one presentation at any academic conference differed significantly among the four types of institutions (*p*=0.03), as shown in Table 2. In contrast, no significant difference was seen in the duration of training among residents at the four types of institutions (*p*=0.10).

**Table 2.** Differences in Research Experience by Institution Type.

	National/public university hospital	Private university hospital	Children’s hospital	Community hospital	*p*
	(n = 499)	(n = 371)	(n = 140)	(n = 490)	
Duration of training in years, Mean ± SD	3.9 ± 1.4	3.9 ± 1.3	3.7 ± 1.1	4.0 ± 1.5	0.08
Experience of presentations at any academic conference, n (%)	460 (92.2)	342 (92.2)	130 (92.9)	429 (87.6)	0.04
Experience of publications, n (%)					
Experience of publishing in any academic journal	135 (27.1)	82 (22.1)	26 (18.6)	84 (17.1)	<0.01
Experience of publishing in peer–reviewed journals	100 (20.0)	56 (15.1)	17 (12.1)	62 (12.7)	<0.01
Peer–reviewed Japanese domestic journals	70 (14.0)	44 (11.9)	8 (5.7)	57 (11.6)	0.06
Peer–reviewed international journals	38 (7.6)	15 (4.0)	10 (7.1)	10 (2.0)	<0.01
Peer–reviewed case reports	74 (14.8)	42 (11.3)	11 (7.9)	50 (10.2)	0.06
Peer–reviewed original articles	27 (5.4)	20 (5.4)	6 (4.3)	19 (3.9)	0.64
Experience of publishing in non–peer–reviewed journals	56 (11.2)	42 (11.3)	10 (7.1)	34 (6.9)	0.05

We found that the proportion of residents who published at least one article in any academic journal and peer-reviewed journals differed significantly among the four types of institutions (*p*<0.01, Table 2). With regard to the types of peer-reviewed journals, the proportion of international journals differed across groups (*p*<0.01). However, no significant difference was found in the proportion of peer-reviewed Japanese domestic journals, case reports, and original articles (*p*=0.06, *p*=0.06, and *p*=0.64, respectively).

We further analyzed the number of presentations and publications by residents by institution type (Table 3). The total number of presentations and the number per year at any academic conference differed across groups (*p*=0.02 and *p*=0.01, respectively). With respect to the number of peer-reviewed publications among institution types, a significant difference was found only in international journals (*p*=0.01).

**Table 3.** Differences in Number of Presentations Given at Conferences and Number of Publications by Institution Type.

	National/public university hospital	Private university hospital	Children’s hospital	Community hospital	*p*
	(n = 499)	(n = 371)	(n = 140）	(n = 490）	
Number of presentations at conferences, Mean ± SD					
Presentations at any academic conference	4.7 ± 3.3	4.4 ± 2.8	4.3 ± 3.1	4.1 ± 3.1	0.02
Presentations per year at any academic conference	1.3 ± 1.0	1.2 ± 0.8	1.2 ± 0.9	1.1 ± 0.9	0.01
Number of publications, Mean ± SD					
Any academic journal	0.4 ± 0.7	0.3 ± 0.8	0.3 ± 0.7	0.4 ± 2.5	0.90
Peer–reviewed journals	0.2 ± 0.5	0.2 ± 0.6	0.2 ± 0.5	0.2 ± 0.7	0.41
Peer–reviewed Japanese domestic journals	0.2 ± 0.4	0.2 ± 0.6	0.1 ± 0.2	0.2 ± 0.6	0.15
Peer–reviewed international journals	0.1 ± 0.3	0.1 ± 0.3	0.1 ± 0.5	0.03 ± 0.2	0.01
Non–peer–reviewed journals	0.1 ± 0.4	0.1 ± 0.4	0.1 ± 0.3	0.2 ± 1.9	0.87

Multivariate logistic regression analyses revealed that residents at community hospitals gave presentations at conferences (odds ratio [OR] 0.56; 95% confidence interval [95% CI] 0.36–0.87) and published peer-reviewed articles (OR 0.53; 95% CI 0.37–0.76) less frequently than those at national/public university hospitals (Table 4). Residents in the Kinki region gave more presentations at conferences (OR 2.13; 95% CI 1.11–4.10 and published more often both in any journal (OR 1.54; 95% CI 1.01–2.33) and in peer-reviewed journals (OR 1.88; 95% CI 1.17–3.02) than residents in the Tokyo area. Although residents in the Tohoku region had more publications in any journal (OR 2.42; 95% CI 1.38–4.24), residents in Kyushu/Okinawa region had fewer publications (OR 0.46; 95% CI 0.26–0.81 in any journal; and OR 0.50; 95% CI 0.25–0.98 in peer-reviewed journals) than residents in Tokyo.

**Table 4.** Multivariate Logistic Regression Analysis of Research Experience.

	Experience of presentation at any academic conference		Experience of publishing in any academic journal		Experience of publishing in peer–reviewed journals	
	OR	95% CI	OR	95% CI	OR	95% CI
Duration of training	0.94	0.83–1.07	0.97	0.88–1.07	0.99	0.89–1.10
Training institution						
National/public university hospital	Reference		Reference		Reference	
Private university hospital	1.18	0.67–2.07	0.87	0.60–1.26	0.87	0.60–1.26
Children’s hospital	1.18	0.56–2.51	0.62	0.37–1.01	0.56	0.31–1.00
Community hospital	0.56	0.36–0.87	0.53	0.38–0.73	0.53	0.37–0.76
Location of institution						
Tokyo	Reference		Reference		Reference	
Hokkaido	1.51	0.49–4.64	1.46	0.69–3.08	1.89	0.84–4.25
Tohoku	1.76	0.64–4.85	2.42	1.38–4.24	1.89	0.98–3.62
Kanto except Tokyo	0.71	0.41–1.22	1.25	0.81–1.92	1.61	0.98–2.63
Chubu	2.00	1.00–4.03	1.53	0.97–2.40	1.54	0.91–2.60
Kinki	2.13	1.11–4.10	1.54	1.01–2.33	1.88	1.17–3.02
Chugoku	1.36	0.52–3.61	1.04	0.51–2.12	1.37	0.63–2.98
Shikoku	1.06	0.34–3.31	1.88	0.86–4.07	1.78	0.74–4.32
Kyushu and Okinawa	0.96	0.51–1.78	0.46	0.26–0.81	0.50	0.25–0.98

## Discussion

This is apparently the first nationwide study in Japan describing in detail the scholarly activities of pediatric residents. We found that these activities consisted mostly of presentations at academic conferences, but that most residents had not published their research. In addition, we showed substantial variations in the scholarly activities of pediatric residents by institution type.

We found that almost all the pediatric residents had given a presentation during their residency. However, of 1,500 participants, only 327 (21.8%) residents published an article during their residency, and most commonly submitted to a Japanese domestic journal and wrote case reports. In addition, 235 (15.7%) residents published in peer-reviewed journals; however, 142 (9.5%) of them published in non-peer-reviewed journals. These findings suggest that residents had difficulty writing research papers regarding what they had originally presented at a conference. Previous studies based on a survey of second- and third-year pediatric residents of 22 American pediatric residency programs and Canadian internal medicine residency programs demonstrated similar findings ^(9), (13)^. In response to this issue, researchers have suggested adopting a systematic strategy including mentorship programs in research and allotting time dedicated to research and/or training in research methods as part of the residency curriculum ^(14), (15)^. These solutions might encourage residents to publish their research on what they had previously presented at conferences.

In general, residents at community hospitals gave fewer presentations and had fewer publications than residents at national/public university hospitals, possibly due to insufficient resources at community hospitals for conducting research. A previous survey illustrating the variety of scholarly activities among pediatric residency programs in the Unites States revealed differences between small/medium-sized programs and large programs in terms of the availability of resources for research. They suggested that offering incentives, such as a mentoring award to the faculty, might help foster the scholarly activities of residents at relatively resource-poor hospitals ^(9)^.

Our study found that pediatric residents in Japan lived in major urban areas. The majority of residents lived near the capital city of Tokyo (39.6%, 595, in the Kanto region including Tokyo) and in the metropolitan areas of west Japan (32.6%, 489, in Chubu and Kinki regions). About one-fourth (24.7%, 371) of the pediatric resident population is concentrated in Tokyo; in contrast, only one-tenth of Japan’s population lives in the same area.

We found significant disparities in the scholarly activities of residents according to the location of their institution. Therefore, a systematic and ongoing strategy is needed to evaluate the academic activities of residents at hospitals that provide pediatric care ^(16)^.

### Strengths and limitations

While most previous studies worldwide on the research activities of pediatric residents were questionnaires targeting program directors or surveys conducted in a limited number of institutions ^(9), (10), (11), (12)^, our database has the largest sample size among studies investigating the research activities of pediatric residents. This fact may strengthen the reliability of our findings.

There are several limitations to this research. First, our board examination system is in the process of development; thus, as explained earlier, not all the participants were actually residents. The generalizability of this study is therefore limited, since the data from logbooks could include research activities after the completion of residency if a participant took the board examination later than usual. However, the average duration of training was 3.9 years, with a small SD (1.4), showing that most of the participants were “conventional” residents. Second, we extracted data on the residents’ research activities from the self-reported logbooks and did not evaluate the confidentiality aspect. Third, it is assumed that scholarly activities of residents are closely related to their workloads, such as the number of patients to care for, duty hours, and number of night shifts; therefore, ideally, achievement in the research activities of residents needs to be adjusted by these indicators for their workload. However, it is not currently possible to access information on pediatric residents’ wellness that is linked to their scholarly activities in Japan. Finally, although we revealed a disparity in the academic activities of residents among different types of institutions, the nature of the barriers to research at institutions with limited scholarly activities and how the faculty there can overcome this issue are unclear. Therefore, another prospective study including qualitative data on the residents and faculty is required to explore solutions to these institutional disparities.

In conclusion, only 16% of pediatric residents in Japan published a peer-reviewed research paper despite the fact that more than 90% gave at least one presentation during their residency. Furthermore, we found variations in the scholarly activities of residents among institution types. A national, systematic approach is needed to promote publications by residents and provide academic mentorship across institution types.

## Article Information

### Conflicts of Interest

None

### Sources of Funding

This work was supported by a grant from the National Center for Child Health and Development in Japan, grant number 26-15.

### Acknowledgement

The authors wish to thank all those who participated in this survey.

### Author Contributions

AI and RM contributed to the conception and design of this study; AI and TK made the database; NM performed the statistical analyses; AI and ON drafted the manuscript; KN and KK critically reviewed the manuscript. All authors read and approved the final manuscript.

The members of Japan Pediatric Society Steering Committee of Board Examination: Satoru Nagata, Tetsushi Yoshikawa, Shinichiro Sekiguchi, Hirokazu Arakawa, Koichi Kusuhara, Katsuhiko Yoshii, Toshiyuki Kitoh, Naoko Ishitoya, Hiroshi Azuma, Junichi Oki, Mika Ishige, Yasuyuki Suzuki, Akiyoshi Nariai, Yasuhiro Takeshima, Kohmei Ida, Kenji Ihara, Masao Kobayashi, Ryuta Nishikomori, Ichiro Morioka, and Masahiko Kishiro.

### Ethical Approval

This study was approved by the Ethics Committees of both the National Center for Child Health and Development in December 2014 (No. 74) and the Japan Pediatric Society in March 2015.
